# Membrane-Based Harvesting Processes for Microalgae and Their Valuable-Related Molecules: A Review

**DOI:** 10.3390/membranes11080585

**Published:** 2021-07-30

**Authors:** Roberto Castro-Muñoz, Octavio García-Depraect

**Affiliations:** 1 Tecnologico de Monterrey, Campus Toluca, Avenida Eduardo Monroy Cárdenas 2000 San Antonio Buenavista, Toluca de Lerdo 50110, Mexico; 2 Department of Process Engineering and Chemical Technology, Faculty of Chemistry, Gdansk University of Technology, 11/12 Narutowicza St., 80-233 Gdansk, Poland; 3 Institute of Sustainable Processes, University of Valladolid, Dr. Mergelina, s/n, 47011 Valladolid, Spain

**Keywords:** algae biomass, harvesting, membrane technology, microalgae biorefinery, renewable energy

## Abstract

The interest in microalgae production deals with its role as the third generation of feedstock to recover renewable energy. Today, there is a need to analyze the ultimate research and advances in recovering the microalgae biomass from the culture medium. Therefore, this review brings the current research developments (over the last three years) in the field of harvesting microalgae using membrane-based technologies (including microfiltration, ultrafiltration and forward osmosis). Initially, the principles of membrane technologies are given to outline the main parameters influencing their operation. The main strategies adopted by the research community for the harvesting of microalgae using membranes are subsequently addressed, paying particular attention to the novel achievements made for improving filtration performance and alleviating fouling. Moreover, this contribution also gives an overview of the advantages of applying membrane technologies for the efficient extraction of the high added-value compounds in microalgae cells, such as lipids, proteins and carbohydrates, which together with the production of renewable biofuels could boost the development of more sustainable and cost-effective microalgae biorefineries.

## 1. Introduction

**Figure 1:**
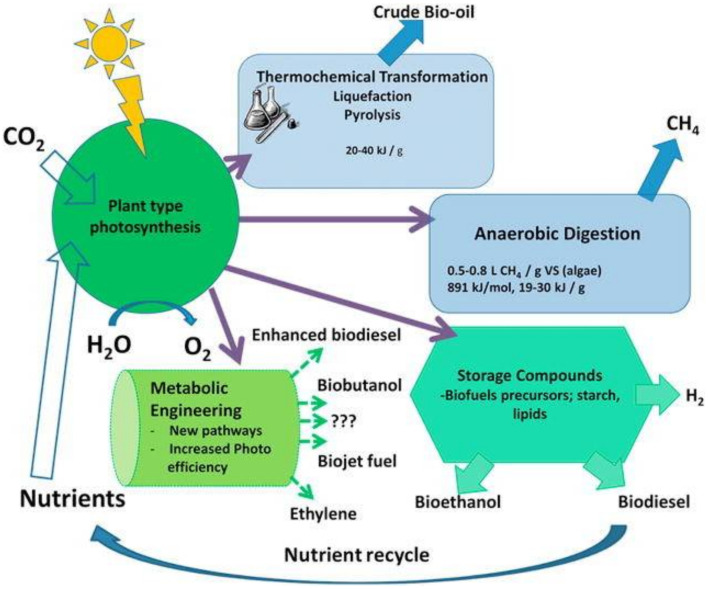
Renewable energy production based on microalgae biomass production. Reprinted with permission from Hallenbeck et al. [5], Copyright 2016, with permission from Elsevier.

Today, there is a big interest in implementing renewable energies as a way of replacing the conventional fossil fuels derived from coal, natural gas and crude oil, which in fact are highly polluting to the environment [1]. The global renewable electricity is produced from renewable sources in which biomass contributes only 1.8%. According to current reports, renewable energy satisfies around 13% of the global energy demand [2]. Microalgae biomass production is a core alternative for the production of renewable energy named as the third-generation feedstock. It is known that microalgae imply multicellular organisms that generally display a fast growth rate at different conditions. Microalgae contains a high content of lipids (mainly triacylglycerides) that represent a feasible feedstock for biodiesel production. Commonly, microalgae contain oil levels ranging from 20 to 50% by weight of dry biomass [3], but higher production rates can be achieved. Additionally, microalgae biomass contains carbohydrates and sugars that can be converted to bioethanol via fermentation [4]. As graphically illustrated in Figure 1, microalgae allow the production of many other renewable energies according to the metabolic pathway of the algae and cultivation conditions.

The production of microalgae becomes more attractive since they can basically grow in harsh conditions (e.g., saline water) with minimal competition of fresh water; moreover, when using closed culture systems, they do not depend directly on the weather conditions, allowing their cultivation during the whole year [5,6]. To date, various strategies are currently used for the production of microalgae, such as photoautotrophic production (open pond production, closed photobioreactors and hybrid systems) [7], heterotrophic production [8] and mixotrophic production [9]. Among all these production pathways, photobioreactor systems stand out as the feasible tool for the production of algae biomass at controlled conditions. It is likely that the main drawback of photobioreactor relies on the diluted concentration of the biomass that oscillates between 0.2 and 0.5%, depending on the types of strain [10,11]. This makes an additional concentration step (well-known as dewatering) needed to reach a final concentration ranging from 15 to 20%.

Importantly, considering the small size of algae cells in the order of micrometers to tens of micrometers, the harvesting and dewatering of the biomass from cultivation media becomes a great challenge. Flocculation, flotation, sedimentation and electroflocculation are among the primary harvesting operations, which display operational issues due to the microalgae in suspension owning a similar density to water [12], while some of them are energy intensive. As a consequence, chemical engineers have implemented secondary harvesting methods, such as centrifugation, rotary filtration, vacuum filtration, direct drying, freeze drying and membrane filtration [13,14]. This latter method implies micro (MF), ultra (UF)-filtration and concentration-driven membrane technologies that have been widely used in the separation, recovery and fractionation of biomolecules from various types of streams, such as wastewaters, by-products, natural sources [15,16,17], among others. In this review, we outline the ongoing research developments at harvesting microalgae using such membrane-based technologies. Additionally, the principles of membrane filtration are given to understand the main parameters that influence on the separation performance. This review also covers the application of membrane technologies for the efficient separation of the specific components contained in microalgae cells, such as lipids (triacylglycerides), proteins and carbohydrates.

## 2. Principles of Membrane-Based Processes and Factors Influencing Their Performance

As in all membrane technologies, the membrane is the main physical element used for the separation of molecules in liquid and gas states. The membrane is defined as a semi-permeable barrier that displays preferential selectivity. According to the principle of membrane technology, the molecules can be successfully separated using the membrane depending on the driving force (e.g., the difference of concentration, pressure and temperature). In the case of pressure-driven membrane technologies, the membrane is able to differentiate between molecules due to their difference in size and molecular weight. The pore size is the main criteria used to categorize MF and UF membranes, as specified in Table 1.

When dealing with microalgae harvesting, MF and UF membranes are likely to be the most investigated by the research community [18]. By applying the driving force, the membrane can split the main feed stream into two different streams, such as permeate and retentate. The first one is majorly composed of the solvent (generally water as the primary solvent in algae cultivation) and all those compounds that were able to pass through the membrane; in other words, the compounds that own a lower molecular weight than the membrane’s cut-off. On the contrary, the retentate has a minor percentage of the solvent, together with all those compounds rejected by the membrane (higher molecular weight than the membrane’s cut-off). In principle, MF membranes, having the wider pore size, are able to retain suspended particles, oil emulsions, specific bacteria and cells and colloidal haze. UF membranes, with a narrower pore size than MF, can collect in the retentate side various molecules, such as viruses, proteins and other macromolecules. Nano (NF)-filtration membranes, together with reverse osmosis, have the tightest pore size; NF membranes can retain sub-molecular organic molecules, divalent ions and low molecular weight compounds (e.g., sugars, polyphenols, etc.). Regarding the operation, these processes could be operated in dead-end or cross-flow (well-known as tangential flow) mode, and both modes can help towards the concentration of algae biomass [7,19]. In the dead-end mode, the feed bulk is fed perpendicularly to the membrane surface; unfortunately, the rejected particles usually form a cake layer on the membrane surface, which represents an additional barrier for the permeating molecules provoking long filtration periods. In this configuration, the operation is also limited by such a cake layer, requiring the batch operation to remove the particles. In the cross-flow mode, the feed bulk is fed tangentially to the membrane surface, where the generated shear forces help to mitigate the formation of a cake layer, thus, enhancing filtration by decreasing the risk of fouling; the cross-flow mode is actually the most applied configuration at large-scale applications.

As reported in Table 2, molecular sieving, based on the difference of size and molecular weight, is recognized as the main separation mechanism in these processes. However, the membrane pore size is not the only parameter that influences on the performance of a membrane, there are also other factors that play an important role in the operation of a membrane process, as follows:

- *Asymmetry properties of a membrane*: In the case of polymer membranes, phase inversion technique is commonly used for the preparation of porous membranes [20]. Such a fabrication protocol often, depending on the type of method and conditions, generates asymmetric properties on the resulting membranes, which means that the membrane does not present a uniform pore size over the membrane structure. Such an asymmetry is a result of handling different parameters, such as exposure time, humidity, polymer concentration, in the preparation protocol. Importantly, an asymmetric structure is the most preferred since it combines high selectivity of small pores in a thin selective layer and high permeability due to low resistance of the support layer.
- *Intrinsic properties of a membrane*: As it is well known, the membranes, depending on the polymer or inorganic material, may either present hydrophilic or hydrophobic properties. In MF and UF processes, hydrophobic materials are preferred (polyethersulfone, polysulfone, etc.) since they repel water molecules, along with all those water-soluble compounds. In addition to this, the surface morphology influences the separation performance, but more importantly, contributes to some specific issues on the operation; for example, the membrane roughness, especially a rougher membrane surface, contributes to fouling. To some extent, the protuberances on a surface allow the capture of organic matter. Here, if there is an accumulation of organic material that may represent a source of microorganism proliferation, the membranes will be susceptible to biofouling formation as well [21].
- *Membrane–molecule interactions*: In general, electrostatic interactions may occur between membrane surfaces and specific solutes that present any charge. Of course, the membrane should also reveal any type of charge, which is often associated with the availability of functional groups on the membrane surface. Eventually, specific solute–membrane interactions, such as the hydrophobic interaction, Coulombic intermolecular attraction and repulsion, are among the most identified forces in membrane processes [22].
- *Membrane fouling*: This factor acts as the main bottleneck of membrane processes since it can lower the flux by pore blocking. The membrane fouling depends crucially on the physicochemical composition of the feed solution to be treated. Here, the possible interactions among the solutes and the membrane can introduce the degree and type of membrane fouling [23]. However, it is worth mentioning that the parameters of the operation may also foster such a phenomenon.
- *Operating parameters*: The permeate flux is usually increased as a function of the driving force; this is possible until the limiting transmembrane pressure is reached [24], in which after such limiting pressure the permeation becomes governed by the fouling and concentration polarization phenomenon. Similarly, the permeate flux can be raised as a function of temperature increase, which is a result of decreasing the viscosity of the fluid and the increasing diffusion of the components. When dealing with fouling issues, the feed flow rate, temperature and transmembrane pressure are important parameters in the membrane fouling. For instance, the feed flow speed influences the shear forces on the membrane surface; as mentioned previously, this generates the partial removal of solutes from the surfaces and, thus, reducing the fouling formation [25]. On the other hand, the retained molecules on the membrane provoke the pressure increment since the fouling layer acts as an additional barrier.

As it can be seen, various factors and parameters play a crucial role in membrane performance once implemented in the membrane process. This becomes more challenging when complex solutions such as microalgae culture are filtrated. Therefore, the following section collects the main strategies adopted by the research community for the harvesting of microalgae. For this, the most relevant outcomes in the field have been discussed.

## 3. Development Works on Membrane-Aided Harvesting Process for Microalgae

Considering various criteria (i.e., energy need, costs, processing time, efficiency, species specific, biomass quantity/quality and toxicity), filtration is considered among the most applicable methods for separating microalgae from their culture medium, even for different applications, including biofuel, human and animal food, high-marketable products and water quality restoration [26,27]. As shown in Table 2, several microalgae strains were harvested using membrane-based processes, for instance, *Scenedesmus acuminatus* [28], *Chlorella zofingiensis* [29], *C*. *pyrenoidosa* [30], *Dictyosphaerium* sp. [31], *Nannochloropsis* sp. [32], *Microcystis* sp. [33], as well as some shear sensitive species such as *Dunaliella salina* [34], *Pavlova lutheri* [35] and many more. The membrane filtration process is chemical-free, not-toxic, can achieve high separation efficiencies (up to 100%) and allows the continuous/discontinuous separation of microalgae and the reusability of the broth [36]. However, the harvesting of microalgae using membrane-based technologies requires a relatively high-energy input which, together with fouling, constitutes the major bottleneck for large-scale applications [28,37]. Membrane fouling caused by blocking, cake formation and/or the adsorption of gel-foulants such as extra- and intracellular organic matter (e.g., proteins, polysaccharides, lipids and humic-like substances) negatively affects the membrane flux, increases maintenance and operating costs, and prevents efficient long-term use [29,36,38]. Therefore, the major goals in membrane-based microalgae harvesting processes are to reduce costs by mitigating fouling; thus, increasing membrane flux and energy consumption efficiency. The intrinsic characteristics of the membrane (e.g., material including additives, surface charge, porosity, hydrophilicity and synthesis method) and filtration unit (e.g., design, operational parameters, operation mode, hydrodynamic and cleaning protocol) employed, along with the physicochemical properties of the microalgal broth (e.g., characteristics of microalgal culture, cell size, density and overall charge, nutrients, organic matter, etc.), greatly impact the capacity, efficiency and cost of the harvesting process. For instance, negatively charged membranes may offer benefits in microalgae filtration [39]; algal species with non-spherical, larger size and a rigid cell often showed an enhanced filtration performance with less algae deposition on the membrane surface [39,40]; unlike low temperatures, high temperatures of the culture broth may induce a lower extracellular organic matter and decline the liquid viscosity which helps to improve the flux through the membrane [41,42]; an increase in culture salinity may reduce the permeate flux rate due to more severe membrane fouling [43]. Thus, efficient fouling control not only requires the synthesis of enhanced tailor-made membrane materials but also of the design, operation, control and optimization of the overall process considering both the microalgal cultivation and harvesting stages. In this regard, different fouling mitigation technologies have been extensively proposed and studied in the last few years, including air-assisted backwashing technology [28,44], dynamic filtration with rotating disks [45] and vibrating systems [31,46,47], flocculation-assisted filtration [48,49], the fabrication of membranes with enhanced antifouling properties by using suitable materials, additives and synthesis methods [32,39,50,51,52], and specific adaptations to the membrane systems, such as electric-assisted forward osmosis [53] jet-assisted MF [54], tilted panel system [55,56].

### 3.1. Air-Assisted Backwashing Technology

**Figure 2:**
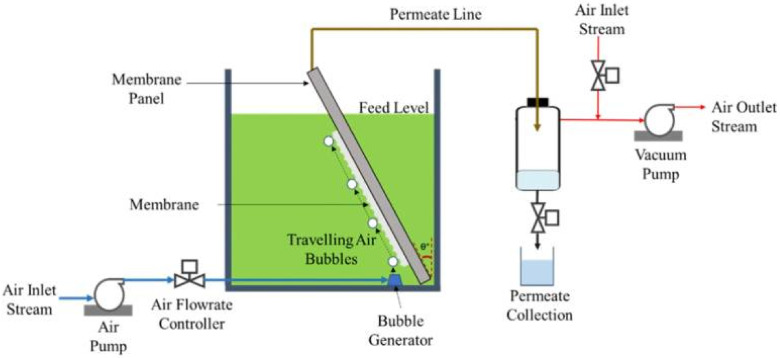
Illustration of a submerged filtration system with a tilted membrane panel used for the harvesting of *Euglena* sp. Reprinted with permission from Lau et al. [56], Copyright 2020, with permission from Elsevier.

In submerged filtration systems, where recirculation provides the cross-flow velocity, membranes are set above micro-porous pipes that generate air bubbles, scouring the foulants along the membrane surface (Figure 2); thus, reducing fouling to some extent [18]. This fouling control strategy majorly depends on the characteristics of the feed, the type and configuration of the membrane panel, as well as the aeration mode and rate. Higher aeration rates normally result in increased permeabilities, albeit the high energy consumption required for pumping may limit the process applicability. It has been shown, however, that air scouring may not be able to deal with more fouled membranes [30,55]. Irreversible fouling occurs mainly at large fluxes and extended filtration times [41]. Likewise, continuous bubbling commonly leads to higher permeation fluxes than the intermittent mode by constantly scouring-off the foulants from the membrane surface; however, under certain circumstances, the intermittent bubbling may outcompete the continuous mode, as found by Nawi et al. [51] who argued that the continuous presence of bubbles might act as a cushion and hinder the contacts of the feed with the membrane. Besides high energy input, the relatively weak shear rates attained in air bubbling scouring systems is also pointed out as one important limitation for the process. In a recent work, Eliseus et al. [44] proposed the use of tilted membranes to improve the contact of air bubbles with the membrane surface. The authors investigated the effect of the tilting angle, switching period (in a two-sided membrane panel) and aeration rate using a tilting panel and compared its filtration performance with that using a conventional vertical panel. Herein, higher tilting angles of up to 20° and aeration rates of 1.8 L/min (equivalent to a specific aeration demand of 0.23 Nm^3^/m^2^ h) resulted in 2.7 times higher permeation rates than those of the vertical panel, while the use of a two-sided membrane did not further improve the cleaning efficiency. Similar filtration behaviors were reported by Lau et al. [56], who reported an outstanding permeability of 724.3 L/m^2^ bar h, when employed a one-sided panel at an aeration rate of 1.8 L/min and a tilting angle of 20°. In other study, Ismail et al. [55] found an optimum tilting angle of 45°, lower or higher tilting angles did not improve the permeability performance, indicating that this type of filtration requires to be optimized with respect to the aeration rate and tilting angle. Besides such factors, membrane properties, mainly the pore size, should be tailored to improve the filterability of microalgae [56]. The effectiveness of air bubble scouring with tilted panels was also recently proved in the harvesting of *Chlorella vulgaris* using an improved nylon 6,6 nanofiber membrane, achieving a permanence and a flux as high as 40.2 L/m^2^ h and 402.3 L/m^2^ h bar, respectively [51]. Other harvesting systems drastically improved membrane performance by increasing hydrodynamic powder with a bubble-generator plate [57]. A novel finned spacer system built into a vertical membrane panel helped to direct air bubbles toward the membrane surface, attaining the highest permeance of 870 L/m^2^ h bar at 1.5 L/m aeration for the harvesting of *Chlorella vulgaris* [58]. Moreover, the finned spacer can be positioned in between two adjacent vertical panels, allowing to operate in the switching mode due to the moving part of the fins, a fact that might make it more reliable and practical than tilted membranes which require the movement of the whole membrane panel to accommodate a suitable position angle.

Membrane cleaning frequency also impacts on the operation flux and fouling. It has been reported that operating in a filtration/backwashing regime may outperform the regular filtration/relaxation operation [35]. The efficiency of backwashing depends mainly on the characteristics of microalgae, the type of membrane and cleaning frequency. Frequent backwashing may help to partially recover and maintain suitable membrane fluxes, but only to a certain extent [40,59]. Too short backflush intervals may not be sufficient enough to control membrane fouling [35] or may provoke cell damage due to the generation of excessive local shear stress and decrease the working time and the amount of the permeate collected [34]. Contrarily, longer backwashing intervals may decrease the efficiency of air-assisted back-washing especially with extended filtration times [28]. It should be noted that severe chemical backwashing is needed to cope with the irreversible fouling, since backwashing using water or air only flushes out the cake layer and part of the particles on the surface (and inside) the pores [29]. The more commonly used membrane cleaning agents are NaOCl, HCl, HNO_3_, and NaOH; however, the unsuitable application of chemicals could deteriorate the membrane or cause cell lysis. Tailor-made back washable membranes have shown a better performance than commercially available membranes, which have also been proven to be efficient for polishing purposes, allowing the recycling of water and nutrients while removing bacteria and algal debris [35]. The operating cost associated with periodic backwashing also deserves a further evaluation.

### 3.2. Dynamic Filtration Systems

In dynamic membrane filtration systems, the hydrodynamic conditions on the membrane surface are altered, by vibrating the membrane instead of the surrounding fluid or by moving a mass at the liquid–membrane interface, in order to achieve a high membrane shear rate and alleviate fouling [18]. Turbulence is produced employing rotating or vibrating systems, or by switching the feed flow direction across the membrane surface [59]. Although dynamic filtration is a complex microalga harvesting process that requires high energy to rotate/vibrate the disks or membranes and equipment cost, the improvement in filterability may lead to a profitable fouling control strategy [60]. For instance, filtration permeability values in shear-enhanced filtration (by vibration) have been reported to be 30–50.3 L/m^2^ h bar, which are 1.5–5 times higher compared with those accommodated by conventional tangential cross-flow filtration [61,62]. Regarding energy requirements, the use of intermittent vibration-assisted filtration has been proposed to reduce fouling while saving more energy than its continuous counterpart (0.21 vs. 9.7 kWh/m^3^) [31]. Using a magnetically induced membrane vibrating system, the energy consumption was estimated as 0.77–0.84 kWh/m^3^, equivalent to 1.39–1.46 kWh/kg of harvested microalgae [63]. Moreover, although the membrane flux, energy consumption, fouling and cell disruption can be tuned and optimize (for example by modulating the frequency, amplitude, cycle time, vibration ratio, etc.) [31], dynamic filtration is difficult to scale-up [62]. Fouling caused by extracellular organic matter and algae debris rather than cell deposition is still challenging in dynamic filtration units even at high surface shear rates [31,46,47,64,65,66]. The use of perforated rotating membrane disks in the dynamic microfiltration of microalgae has been also proven to increase the shear stress of the fluid on the membrane surface, doubling the permeate flux (381 L/m^2^ h) during the harvesting of *Chlorella vulgaris* in comparison with that exhibited by a shear-enhanced microfiltration system equipped with unperforated disks as the control [45]; indeed, orifice-based shear rate generation has been reported as a successful means to prevent fouling, sustaining fluxes as higher as 104.5 L/m^2^ h [67].

**Figure 3:**
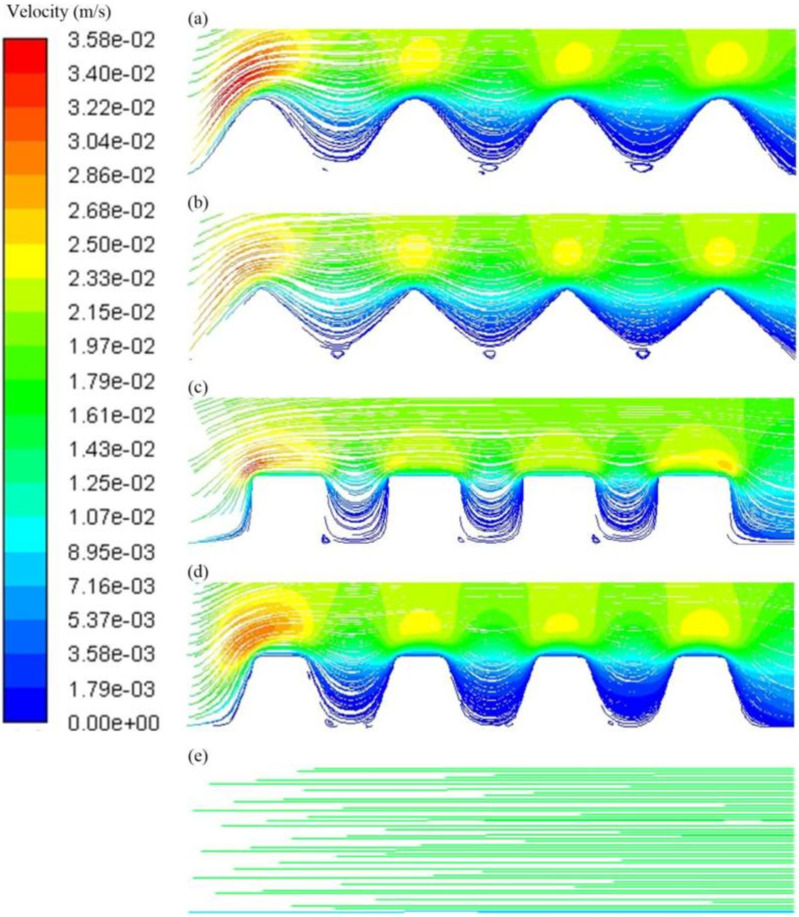
Comparison of the velocity streamline profiles near the membrane surface simulated by computational fluid dynamics (CFD) simulation of (**a**) wave, (**b**) triangle, (**c**) rectangle and (**d**) trapezoid patterned membranes vs. (**e**) a flat membrane at Reynolds number *(Re*) = 109. Red and blue colors represent higher and lower velocities, respectively. In the cases where a patterned membrane was used (**a**–**d**), the vortices formed in the bottom of the valley region may alleviate microalgal cell deposition. In contrast, the flat membrane exhibited a constant low-flow behavior which may not be high enough to reduce fouling. Reprinted from Zhao et al. [69], Copyright 2021, with permission from Elsevier.

Membrane surface patterning has been also employed as an alternative to tackle membrane fouling (Figure 3), enhancing membrane fluxes and, therefore, decreasing total microalgae harvesting costs [52]. In patterned membrane, it is possible to tune new geometry prism patterns, such as waves, triangles, rectangles, trapezoids, in order to enhance the formation of a vortex and reduce the portion of the permeation stream in the valley region, and, thus, resulting in the mitigation of particle deposition on the surface membrane (Figure 3) [68,69]. Very recently, Zhao et al. [70] evaluated the synergy between the flocculation and patterned membrane using polysulfone and *Dictyosphaerium* sp. as the membrane material and model microalgae strain, respectively. This strategy exhibited enhanced antifouling properties (which might improve membrane lifetime) due to the increase in the filtration active area per m^2^ of the membrane and the cross-flow behavior, particularly, the enhancement of local turbulences near to the membrane surface; therefore, ensuring a very high membrane permeances as high as 110 L/m^2^ h bar using a low cross-flow velocity of 0.0025 m/s. The energy inputs and total harvesting costs were estimated as 0.28 kWh and EUR 0.16 per kg of harvested microalgae. In flocculation-assisted membrane systems, the flocculation step needs to be optimized as it can affect membrane permeance and harvesting efficiency; optimized flocculant type and dossing will result in an improved filterability by giving bigger flocs and less extracellular organic matter content [48,71]. Previous studies suggested that the coagulant/flocculant type and dossing are species and membrane specific [33,48,49]. Chitosan is one of the most used flocculants, but its price significantly contributes to the total harvesting cost [70]. Electrostatic interactions between the microalgae and the surface of the membrane also impacts on fouling [72]. Introducing surface negative charge in a wave-patterned membrane was recently reported to be an effective approach to alleviate fouling, exhibiting 100% harvesting efficiency of *Desmodesmus* sp. with membrane permeances of up to 1000 L/m^2^ h bar [69].

### 3.3. Membrane Manufacture

**Figure 4:**
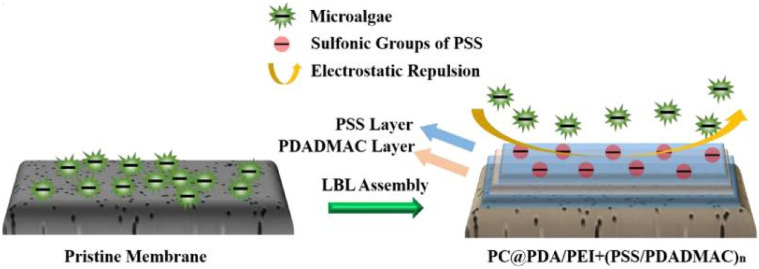
Schematic illustration comparing the antifouling mechanism of a neutrally charged pristine polycarbonate (PC) membrane and a charged and hydrophilic PC membrane fabricated using the layer-by-layer (LBL) self-assembly technique with polydopamine (PDA) and polyethylenimine (PEI) coating and further uniform assembling of poly-(styrenesulfonate) (PSS) and poly-(diallyldimethylammonium chloride) (PDADMAC) via electrostatic attraction. The cell surface charge of most microalgae is (slightly) negative [36]. Reprinted with permission from Huang et al. [78], Copyright 2020, with permission from Elsevier.

Regarding the materials used for the fabrication of membranes, the ideal membrane should pose a robust mechanical strength, high permeability, excellent chemical properties (e.g., compatibility, acid, alkali and chlorine resistance) and low investment costs [28]. Polyvinylidene difluoride (PVDF) has long been proven to be a promising base polymer for harvesting microalgae due to its high thermal/chemical resistance and tensile strength [73]. Low-cost ceramic membranes can also offer thermal and chemical stability as well as a high mechanical strength [74]. However, more studies on the evaluation of the performance of ceramic membranes for microalgae harvesting are still needed. Structural properties of the membrane are commonly characterized in terms of morphology, pore size, porosity, surface free energy, zeta potential, bulk composition, surface composition, wettability, and surface pore area [73,75], while its performance is assessed by determining the flux (including clean water permeance), fouling resistance, and harvesting efficiency in terms of the concentration factor, volumetric reduction factor, recovery rate and cell viability. Higher hydrophilicity and negative charge are properties sought toward the design of anti-fouling membranes, in part, due to the cell surface charge of most microalgae being (slightly) negative [36]. An increased hydrophilicity and negative charge have been obtained by incorporating sulfonated polysulfone to the membrane [69]. Low-fouling composite membranes with enhanced superhydrophilicity and underwater superoleophobicity properties have been proven to reduce algae deposition on the membrane surface [76]. Nylon 6,6 nanofiber [51,77] and polyacrylonitrile [39] membranes have been tested in the membrane-aided harvesting process for microalgae with encouraging results. The layer-by-layer self-assembly technique has been employed to modify a neutrally charged polycarbonate membrane cross-linked with a polydopamine and polyethylenimine coating, creating a high negatively charged and hydrophilic membrane with anti-fouling properties for microalgae filtration, as shown in Figure 4 [78]. Negatively charged membranes were fabricated using polysulfone blended with sulfonated polysulfone in dimethylacetamide [50] and hydrophilic polyvinyl alcohol polymer [79]. Another study reported the blending of polyethersulfone (PES) polymer with multiwall carbon nanotubes and lithium bromide salts in dimethylacetamide via thermally induced phase separation; the resulted membrane was tested for *Nannochloropsis* sp., showing good anti-fouling properties with enhanced hydrophilicity [32].

Besides membrane material, membrane pore size greatly affects the harvesting performance; thus, its adequate selection is of utmost importance to reduce fouling risk [30]. The size of a membrane should be large enough to allow high permeance but small enough to retain the microalgal cell without blocking the pore [48]. According to Gerardo and coworkers, the influence of pore size on the microalgae harvesting performance remains inconclusive [80]. MF and UF are the most used membrane filtration methods for microalgae harvesting, albeit forward osmosis has also been applied for microalgae harvesting [81]. It is noteworthy to mention that the fact that a given membrane exhibits high water permeability does not necessarily mean that it can sustain high microalgae permeability. UF commonly outcompetes MF (in terms of permeate flux, algae cell retention, and fouling resistance), since the latter is more susceptible to intrapore fouling because of its larger pore sizes [34,35,60,82]. However, sometimes MF may perform better than the UF process depending on the hydrodynamic conditions and algae culture characteristics, as concluded by previous works [34,57,83]. In UF technology, it has been shown that a higher pore size can mitigate membrane fouling by decreasing membrane hydraulic resistance (low permeate drag force), which means a reduction in the speed at which algal biomass moves towards the membrane surface [30]. A pore size around 0.1 µm has been found to be adequate for algae harvesting [30,56], which can be modulated by varying the concentration of the polymer, evaporation time, water addition and the concentration of the additive [48]. Altogether, the most suitable membrane pore size should be determined case by case considering both the type of algae species and the membrane process set-up.

### 3.4. Emerging Membrane-Based Microalgae Harvesting Technologies

**Figure 5:**
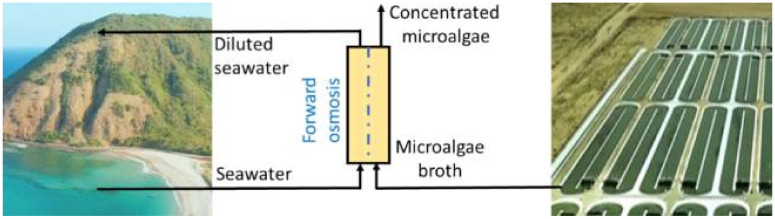
Schematic representation of forward osmosis process using seawater draw solution for the dewatering of fresh water microalgae. Reprinted with permission from Nawi et al. [88], Copyright 2020, with permission from Elsevier.

**Figure 6:**
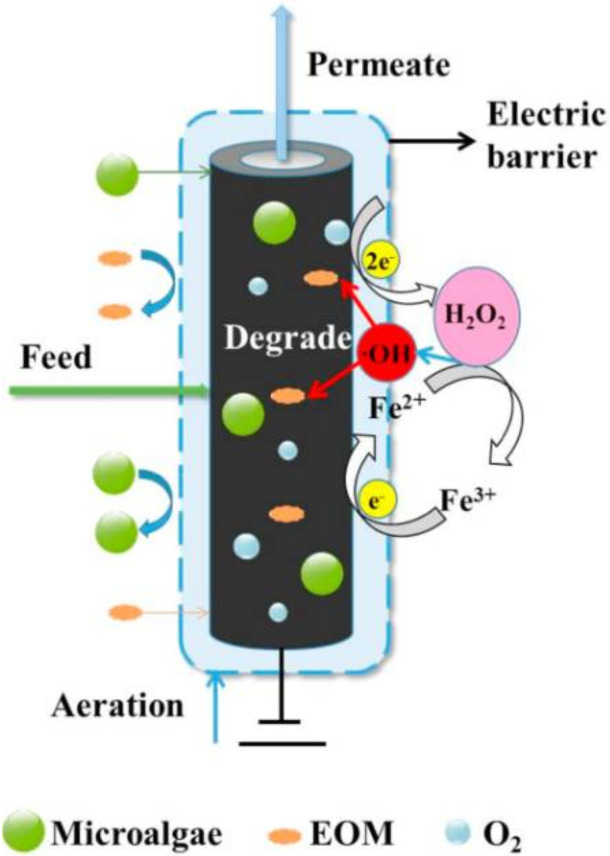
Electro-Fenton-assisted porous carbon–carbon nanotubes–polyvinyl butyral hollow fiber membrane loaded with Fe^2+^ proposed to control fouling caused either by cell deposition or extracellular organic matter (EOM) during microalgae harvesting. Reprinted with permission from Zheng et al. [90], Copyright 2021, with permission from Elsevier.

Forward osmosis has been also applied for the harvesting of microalgae, mainly as an initial dewatering step [84,85,86,87]. Forward osmosis may reduce the cost of harvesting (low-energy consumption) by replacing external hydraulic pressure with the osmotic pressure gradient as the driving force to concentrate microalgae. Hafiz et al. [84] performed a comparative analysis in the performance of a hybrid ultrafiltration–forward osmosis system and a dual-stage ultrafiltration system for the harvesting of *Tetraselmis* sp. The results showed that, although both evaluated systems mediated similar total concentrations factors of 37.3, the use of forward osmosis as a post-harvesting process resulted in 24% less energy consumption compared to the dual stage ultrafiltration process [84]. Compared with pressure-driven membrane processes, forward osmosis may be more conducive to preserving microalgae cell integrity, and also may require less chemical cleaning demand; however, this technology still exhibits low dewatering rates in the range of 1.8–5.6 L/m^2^ h [86,87]. The selection of an appropriate draw solution is of utmost importance in the process. In this regard, various draw solutions have been tested in terms of water and reverse salt fluxes, including NaCl, KCl and NH_4_Cl [87]. Moreover, an increase in the feed solution concentration, concentration polarization, and the attachment of microalgae on the membrane surface have been found to result in a loss of flux in continuous long-term filtration experiments [87]. Using seawater as the draw solution (Figure 5), an aeration-aided forward osmosis process showed water fluxes of around 6 L/m^2^ h with associated volumetric concentration factors of up to 6× [88]. A concentrated heterotrophic microalgal biomass was achieved using glucose-driven forward osmosis, which was able to sustain good dewatering performance (biomass was concentrated from 30 to 120 g/L) by decreasing the reverse solute flux, even at lower values than sea salt did [89]. Other strategies such as electro-Fenton-assisted membrane filtration [90], electrically assisted forward osmosis [53], and turbulent jet-assisted microfiltration [54] have been proposed to reduce reversible and irreversible fouling during the harvest of microalgae. In an effort to cope with uncharged organic matter present in the broth and reduced energy requirements, Zheng et al. [90] evaluated electro-Fenton-assisted porous carbon–carbon nanotubes–polyvinyl butyral hollow fiber membranes loaded with Fe^2+^ to harvest microalgae (Figure 6); the membranes had a pore diameter of 207 nm and supported a clean water permeance higher than 2000 L/m_2_ h bar. The anti-fouling system at an optimized electric field of −1.0 V showed a good harvesting performance (2.5 higher concentration factor, the ratio of feed volume to retentate volume, compared with the control without Fenton reactions, i.e., 4.0 vs. 1.6) due to the in situ hydroxyl radical (•OH) generation which can generate electrostatic repulsion excluding away negatively charged microalgal cells and organic matter from the membrane surface and degrade selectively extracellular organic matter, including proteins, polysaccharides and hydrophobic humic-like substances, on the surface of the membrane and inside the pores. Moreover, a flow cytometry analysis revealed a 3.8% reduction in live cells after a 3 h filtration, mainly due to the fact that hydroxyl radical is highly selective to organic matter and diffuses slowly through the algal membrane, suggesting that the electro-Fenton system may slightly affect the microbial activity. On the other hand, Kim et al. [54] evaluated the use of submerged turbulent jets in a hollow membrane to generate locally high velocity and shear stress near the membrane. Using this strategy during the filtration of *Chlorella*, less cake formation was observed and pore-clogging without deteriorating the integrity of the cells.

### 3.5. Pilot-Scale Studies

Experiences gained from pilot-scale membrane microalgae harvesting are needed to provide a solid proof of its technical viability for commercialization and datasets for future economic–environmental assessments [60]. Unlike bench-scale filtration, in large-scale membrane units the effect of other factors, such as factor concentration and microalgal suspension characteristics such as the growth of undesirable co-cultures (e.g., microzooplankton contamination), must be well understood to develop efficient industrial processes [28]. For instance, permeability values in UF units have been reported to be 30–40% lower when microalgal biomass was cultured in 9000 L outdoor photobioreactors (as compared to those observed when it was growth at lab-scale conditions) due to a higher cell concentration and culture contamination issues [61]. Tangential flow filter membrane [43,91] and dynamic filtration [62] configurations have been tested at a pilot scale. A techno-economic evaluation at pilot scale was carried out by Wang et al. [28] setting an annual harvesting capacity of 10,000-ton dry microalgal biomass. The analysis considered different equipment (i.e., membrane modules, valves, pipelines, pumps, among other accessories) and operating costs (i.e., labor cost and the energy consumption for harvesting, cleaning, backwashing and the transfer of raw water), estimating a total harvesting cost of USD 0.30/kg dry microalgal [28]. The energy cost associated with the filtration of microalgae is variable depending on several factors, and has been estimated as 4.2 kWh/kg or 0.87 kWh/m^3^ [34].

## 4. Membrane Technology for the Downstream Processing of Valuable Products Derived from Microalgal Biomass

Microalgae biorefinery aims at coupling the cultivation of microalgal biomass with the production of biofuels and high-value co-products such as pigments, proteins, lipids, carbohydrates, vitamins, anti-oxidants, among others, which have application in several sectors, including food, cosmetics, as well as pharma industries [92,93]. However, membrane technology for the recovery of marketable commodities from microalgae is still in its learning curve [80]. The most relevant aspects related to the recovery of high-value algal chemical products by membrane-based processes are discussed in the following sub-sections.

### 4.1. Algal Protein Recovery by Membrane Technology

Membrane filtration is seen as a promising method for the recovery of proteins from microalgae not only because of its green nature (since no contaminating chemicals are required), but also because it may preserve both their functional and nutritional properties, which become particularly relevant in the production of health beneficial products [94,95]. The recovery of target proteins usually involves the use of multi-step membrane processes to achieve a high yield and purity. Marcati et al. examined the recovery of B-phycoerythrin from *Porphyridium* using a Cogent M1 pilot-scale tangential-flow filtration unit configured to operate in an ultrafiltration and diafiltration mode using PES flat membranes [96]. By applying the two-step membrane process, it was possible to recover up to 48% of the protein with a purity ratio of 2.3. Safi et al. [97] also applied a two-step filtration (ultrafiltration/diafiltration) process with different membrane cut-offs (1000 kDa, 500 kDa and 300 kDa) to obtain an enriched fraction of water-soluble proteins from *Nannochloropsis gaditana*, which had an initial biomass concentration of 100 g/L. The lowest membrane cut-off led to the highest protein yield of 25%. By contrast, the filtration process was not improved by further increasing the cut-off of the membrane due to adsorptive fouling of polysaccharides.

A proof-of-concept study for the recovery, purification and concentration of proteins derived from microalgal biomass (*Chlorella sorokiniana*) was recently reported using a three-step membrane filtration process encompassing a prefiltration step (ceramic membrane, 0.22-μm) to remove cell fragments, followed by diafiltration (ceramic membrane, 0.22 μm) to recover the proteins previously retained and, finally, a concentration step (ceramic membrane, 3 kDa) in which the proteins are concentrated while washing out salts and small sugars [98]. In that process, the protein recovery yield was 12%, implying that it requires further improvement; the reduction in protein loss caused by degradation and/or adsorption processes, the increase in protein solubility and the enhancement of fouling control were the main challenges that need to be tackled in the future.

In another study, Böcker et al. isolated proteins with emulsification potential from *Arthrospira platensis* using tangential-flow diafiltration. Utilizing a Vivaflow 200 Hydrosart membrane with a 5 kDa molecular weight cut-off, the final protein recovery yield of 11.7% was attained, of which phycocyanin accounted for around 33%. Despite the low yield achieved, the purification process improved the protein’s functionality compared to that of crude protein extracts, this was attributed to the removal of impurities such as surfactants [99]. Food grade phycocyanin (82% yield with a purity ratio of ~1.0 and a concentration of 6.7 mg/mL) from *Spirulina* sp. has been previously obtained by coupled microfiltration and ultrafiltration [100]. More recently, a two-step hydrophobic interaction membrane chromatography was proposed for the first time to purify phycocyanin from *Arthrospira platensis,* yielding 67.0% and a purity index of 4.20 with a commercial hydrophilic PVDF membrane (0.45 µm pore size) [101]. The PVDF membrane showed very low protein binding capacity, minimizing unspecific protein binding on the membrane while allowing its reusability through multiple filtration cycles. Moreover, the membrane was able to retain—selectively and reversibly—phycobiliproteins (which have shown health benefits such as antioxidant, anti-inflammatory, anti-cancer, and anti-viral activities [95]) via a tailored ammonium sulphate precipitation. Ultrafiltration in the diafiltration mode assisted with ammonium sulphate precipitation has also been reported for the purification of R-phycoerythrin from *Gelidium pusillum* (68% yield and a purity index of 0.49), which has a market value of 180–250 USD/mg and commercial applications in immunology, diagnostics, cosmetics and foods [102]. Finally, PES membrane with a molecular cut-off of 30 kDa was effective in recovering R-phycoerythrin from *Grateloupia turuturu*, obtaining a purity index of 1.07 despite the use of a single ultrafiltration step [103].

### 4.2. Application of Membrane Filtration to Recover Algal Exopolysaccharides

In the case of algal exopolysaccharides (EPS), *Porphyridium* is one of the leading microalgae genera used as a source of EPS having nutraceutical and pharmaceutical activities [104]. Indeed, *P*. *cruentum* has been exploited for the production of EPS, and other valuable compounds such as pigments, at large scale [105]. Lab-scale UF using a flat PES membrane with a molecular weight cut-off of 50 kDa was employed to recover EPS produced by the red algae *P. cruentum* [105]. The volume reduction ratio achieved was 10 with a permeation flux of 32 L/m^2^ h. It is important to note that EPS solutions have a strong fouling capacity, even at diluted concentrations, because of their ability to form highly viscous gels and compacts deposits on the membrane’s surface. At a low EPS concentration of 0.1 g glucose equiv./L, irreversible and reversible fouling was ascertained as 88 and 12%, respectively.

It has been reported that carbohydrates lead to a higher fouling risk than proteins when hydrophilic polyvinylchloride (PVC) membranes in ultrafiltration configuration were tested [29]. However, strategies to cope with membrane fouling deserve further research considering the interactions not only between membrane and EPS, but also proteins, lipids and other foulants. EPS from *P*. *cruentum* has been also extracted using cross-flow filtration in the diafiltration mode with a 300 kDa molecular weight cut-off, but with an EPS loss in the permeate of 34% [106]. Diafiltration using a 0.14 μm ceramic membrane sustained mean permeate fluxes between ~50 and 82 L/m^2^ h at four bar depending on the cross-flow velocity, recovering more than 80% of the EPS in a concentrated fraction (6.3 to 10.4×, sugars concentrations of 1.74–2.26 g/L) [107].

Furthermore, the culture conditions (e.g., cultivation mode, irradiance, salinity, etc.) and the physiological state of microalgae affects the profile and concentration of EPS, a fact that contributes to make the extraction of microalgae EPS complex. The concentration of EPS in the culture medium typically reaches low values ranging from 0.1 to 1.0 g/L, which increases the downstream processing cost [105,108]. Thus, the downstream of EPS solutions via membrane filtration is quite challenging. Recently, a pilot-scale cross-flow ultrafiltration system was tested for the recovery of microalgal EPS with potential antitumor activity [71]. In that study, the EPS were isolated from six different microalgal species, including *Nostoc sphaeroides* and *Haematococcus pluvialis*, achieving average permeate fluxes close to 37 L/m^2^ h at a transmembrane pressure of 0.5–0.6 bar and ambient temperature (28–37 °C) regardless of the microalgal culture; however, the EPS varied in the range of 3.5 up to 231.3 mg/L depending on the culture. In another study, EPS from *Porphyridium* were successfully extracted using PES flat UF pilot membranes, obtaining 80% of the initial EPS content after ultrafiltration and diafiltration steps [96]. Altogether, the results of the previous studies show the potential of membrane technology for EPS recovery from microalgae.

### 4.3. Recovery of Lipids

Regarding microalgae lipids, they can be used not only as a feedstock for the production of biodiesel, but also in cosmetics [104,109]. In membrane filtration processes intended to recover lipids from microalgae extracts, it is of utmost importance to choose membrane materials with a suitable size distribution and degree of hydrophilicity since both determine the water and oil droplet permeation. However, too small membrane cut-offs may be more conducive to adsorption pore blocking or cake formation. Hydrophobic membranes may also promote fouling during the filtration of microalgae extracts with a high lipids content [110,111]. Lipid recovery efficiency varies in a wide range from 3 up to 98% depending mainly on the extraction process (e.g., reverse osmosis, dynamic filtration and cross-flow filtration), type and characteristics of the membrane used (e.g., polyimide, polysulfone and polyacrylonitrile) and operating conditions [112]. Villafaña-López et al. evaluated four different commercial membranes for the concentration of lipids from model and real aqueous extracts of *Parachlorella kessleri* [111]. The authors also compared the oil separation performances of cross-flow and rotating-disk dynamic filtration units. The results obtained showed that a commercial polyacrylonitrile membrane (500 kDa) exhibited the best operation performance in terms of oil retention, water permeation and cleanability. The shear-enhanced filtration also performed better than the cross-flow system, supporting the full retention of lipids with minimal fouling issues, even when it filtered real microalgae extracts.

## 5. Conclusions and Perspectives

This review paper denoted the ability of membrane techniques in the harvesting of a microalgae biomass, as well as key principles and factors involved for the successful operation of membranes in such a complex system (culture medium). By analyzing the ongoing advances on membrane-based harvesting processes, this paper identified the main strategies followed by the researchers in collecting the algae biomass overcoming the main bottlenecks in the operation. To date, plenty of microalgae strains have been successfully harvested using membrane-based operations, including *Scenedesmus acuminatus*, *Chlorella zofingiensis*, *C*. *pyrenoidosa*, *Dictyosphaerium* sp., *Nannochloropsis* sp., *Microcystis* sp., along with shear sensitive species such as *Dunaliella salina*, *Pavlova lutheri*, among others. The average separation efficiency has been reported as high as 100%.

In these applications, membrane fouling is still addressed as the most critical factor that compromises the performance of membranes and their continuous operation. Herein, the researchers are strongly working at modifying the membrane surfaces to reduce the membrane fouling, and it seems to be that a future direction will deal with the chemical modification of membranes to obtain highly hydrophilic membranes. As a recommendation to the new researchers in the field, even though that the recovery efficiencies and permeation fluxes seem to be high, future works should be aware of implementing membrane processes adapted with other technologies to guarantee long-term operation. In this regard, future works can give us an overview if such maintenance and operating costs can be reduced. Furthermore, the influence of operating parameters on the harvesting of microalgae at pilot scale deserves further attention.

Finally, the application of membrane techniques not only deals with the harvesting of algae biomass, but also with the recovery of valuable molecules either contained or derived from algal biomass. Specific biomolecules, such as B-phycoerythrin, proteins (e.g., phycocyanin), exopolysaccharides and lipids, can be separated from the culture media. As a concluding remark, the selection of the membrane types and operating parameters will mainly dictate the efficiency of the recovery process; however, the strategy proposed by the researchers will also contribute to the successful separation. In this way, depending on the target molecules, the membrane process design should be smartly proposed. Finally, it is of note that although membrane-based microalgae harvesting processes have become a promising technology, to date, however, it seems that the third-generation renewable energy production alone is uneconomical, but the development of microalgae biorefineries devoted to the co-production of biofuels and value-added fine chemicals could be a more sustainable and cost-effective approach. Thus, there is a gap in the design and evaluation of multi-objective approaches to simultaneously recover microalgal biomass and its related by-products by membrane technology, including its techno-economic assessment.

## Figures and Tables

**Table 1:** Categorization of pressure-driven membrane technologies, their pressure requirements and separation mechanism.

Process	Pore Size (nm)	Pressure Requirement (bar)	Separation Mechanism
MF	100–10,000	0.1–2	Molecular sieving
UF	2–100	0.1–7	Molecular sieving
NF	0.5–2	3–25	Sieving/molecular interactions

**Table 2:** Latest works on microalgal membrane harvesting.

Microalgae	Technology	Membrane	Average Membrane Flux (L/m^2^ h)	Biomass Recovery	Total Harvesting Cost/Energy Consumption	Ref.
*Scenedesmus acuminatus*	Cross-flow UF air-assisted backwashing system (53-m^3^ pilot scale)	Polyvinylchloride (PVC) hollow fiber membrane (cut-off 50 kDa)	56	93% (concentration factor of 145 and final dry weight of 136 g/L)	USD 0.30/kg dry biomass	[28]
*Dictyosphaerium* sp.	Magnetically induced membrane vibration system	12% polyvinylidene difluoride (PVDF) Mw ~543 kDa	46	Harvesting efficiency higher than 97%	0.21 KWh/m^3^	[31]
*Nannochloropsis* sp.	Cross-flow UF	Antifouling Polyethersulfone (PES) membrane with carbon nanotubes and lithium bromide	28.9	100% harvesting efficiency, final concentration of 28 g/L	-	[32]
*Dunaliella salina*	Cross-flow UF	PES capillary membrane (cut-off 150 kDa)	31	Concentration factor of 5.9	-	[34]
*Picochlorum* sp. (*Tetraselmis* sp.)	Pilot-scale cross-flow	Polyacrylonitrile (PAN) hollow fiber (weight cut-off 10 kDa)	37.7 (33.8)	Final concentration of 28 g/L 27.1 g/L (22.0 g/L)	1.81 kWh/m^3^ (3.3 kWh/m^3^)	[43]
*Dictyosphaerium* sp. (*Chlorella vulgaris*)	Dynamic filtration combined with flocculation	PVDF-12% (0.013 µm)	78 (85)	-	-	[49]
*Chlorella vulgaris*	Tilted panel NF	Treated nylon 6,6 nanofiber	37.9	379.5 L/m^2^ h bar	-	[51]
*Chlorella vulgaris*	Turbulent jet-assisted MF	PVDF hollow fiber membrane (0.2 µm)	104	-	-	[54]
*Spirulina* sp.	Tilted panel MF	PVDF (0.42 µm)	55.4	554 L/m^2^ h bar	0.20 KWh/m^3^	[55]
*Chlorella* sp.	Cross-flow MF with a bubble-generator plate	PVDF (0.2)	-	105 L/m^2^ h bar, 100% harvesting efficiency, 1.3 concentration factor	-	[57]
*Chlorella vulgaris*	Submerged filtration system	Pristine nylon 6,6 nanofiber	28.6	286 L/m^2^ h bar	4.16 KWh/m^3^	[58]

## Data Availability

Data are contained within the article.
